# Complete Molecular and Immunoprotective Characterization of *Babesia microti* Enolase

**DOI:** 10.3389/fmicb.2017.00622

**Published:** 2017-04-11

**Authors:** Xiangye Liu, Chen Zheng, Xiaoge Gao, Jiaxu Chen, Kuiyang Zheng

**Affiliations:** ^1^Jiangsu Province Key Laboratory of Immunity and Metabolism, Department of Pathogenic Biology and Immunology, Xuzhou Medical UniversityXuzhou, China; ^2^Jiangsu Center for the Collaboration and Innovation of Cancer Biotherapy, Cancer Institute, Xuzhou Medical UniversityXuzhou, China; ^3^National Institute of Parasitic Diseases, Chinese Center for Disease Control and Prevention, Key Laboratory of Parasite and Vector Biology, Ministry of Health of China, WHO Collaborating Centre for Malaria, Schistosomiasis and FilariasisShanghai, China

**Keywords:** *Babesia microti*, enolase, surface expression, plasminogen, immunoprotective

## Abstract

The apicomplexan *Babesia microti* is the primary causative agent of human babesiosis, one of the most broadly distributed tick-borne diseases worldwide. *B. microti* undergoes a complex lifecycle within both the mammalian host and the tick vector, and employs several different specific molecular mechanisms to enter host cells. Enolase, the key glycolytic enzyme in intracellular glucose metabolism, can also be expressed on the parasite’s outer surface, binds to human plasminogen, and coordinates apicomplexan parasite invasion of host cells, however, it lacks sorting sequences or lipoprotein anchor sites. In the present study, we isolated the coding gene of *B. microti* enolase (BmEno), expressed it within *E. coli* and purified the recombinant BmEno protein (rBmEno). Consequently, we confirmed cytoplasmic and surface localization of BmEno via immunofluorescence, and demonstrated that rBmEno catalyzes the dehydration of 2-phospho-D-glycerate to phosphoenolpyruvate. Moreover, our results showed that rBmEno binds to human plasminogen, and that the lysine analog ε-aminocaproic acid significantly inhibited this binding. Furthermore plasminogen bound to rBmEno converts to active plasmin. Additionally, actively immunizing mice with rBmEno could evoke a partial protective immunity against *B. microti* infection following challenge. In conclusion, *B. microti* enolase is a multifunctional cytoplasmic protein which is also expressed at the parasitic outer surface, facilitates binding to host plasminogen, and could partially protect hosts against parasite infection.

## Introduction

Human babesiosis, caused by the intraerythrocytic apicomplexan parasite babesia, is an emerging infectious disease which manifests worldwide, including the Americas, Europe, Africa, and Asia (Vannier and Krause, 2012). Humans are most commonly infected via bites from ixodid ticks, the predominant Babesia vector; however, blood transfusions and trans-placental transmission can also play important roles in the infection process (Vannier and Krause, 2012). Following infection, the patient usually presents as asymptomatic or with non-specific symptoms such as fever, often in conjunction with fatigue, chills, sweats, headache, myalgia, arthralgia, and/or anorexia, which may include thrombocytopenia, hemolytic anemia, and/or elevated liver enzyme levels (Sanchez et al., 2016).

Babesia is one of the most important tick-borne pathogens in terms of public health, and has a complex lifecycle with developmental stages occurring both within the mammalian host and the tick vector (Vannier and Krause, 2012). As one of its critical developmental steps initially takes place within mammalian host red blood cells (RBCs), the parasite has developed specialized methods to enter RBCs (Lobo et al., 2012). Similar to other apicomplexan parasites, Babesia invades RBCs via the coordinated secretion of several molecules including micronemes, rhoptries, and dense granules from their specialized secretory organelles (Tyler et al., 2011; Lobo et al., 2012). Babesia then completes its life cycle within tick vectors to enable effective transmission. The parasite migrates across the tick gut barrier into the hemolymph via unknown mechanisms, and then into the tick salivary glands (Liu and Bonnet, 2014). Several tick-expressed molecules have been identified which protect the tick against Babesia invasion in the gut epithelium (Tsuji et al., 2007, 2008), however, to the best of our knowledge, no Babesia molecules orchestrating tick gut epithelium invasion have been identified, until now.

A number of vector-borne pathogens express special proteins, such as enolase, on their cell surface to coordinate vector invasion (Pal et al., 2004; Ghosh et al., 2011; Toledo et al., 2012). Enolase is the key glycolytic enzyme in intracellular glucose metabolism, and catalyzes the reversible dehydration of 2-phospho-D-glycerate (2-PGA) to phosphoenolpyruvate (PEP) in the penultimate step of glycolysis (Pancholi, 2001; Diaz-Ramos et al., 2012). Moreover, enolase has been reported to be expressed on the surface of a variety of eukaryotic cells, even though it lacks *trans*-membrane domains or signal peptides (Pancholi, 2001; Diaz-Ramos et al., 2012). Surface expressed enolase has been identified as a plasminogen receptor, which can be subsequently converted into active plasmin, a strong serine protease facilitating the host invasion process (Pancholi, 2001; Ghosh and Jacobs-Lorena, 2011; Diaz-Ramos et al., 2012). In recent years, a variety of pathogenic parasites have also been found to express enolase on their surface, and it is thus thought to aid with host invasion (Figueiredo et al., 2015; Alves et al., 2016; Mahana et al., 2016). Remarkably, enolase has also been identified on the surface of vector-borne pathogens (e.g., *Plasmodium falciparum* and *Borrelia burgdorferi*), and plays an important role during pathogen invasion of vector gut by binding mammalian plasminogen (Ghosh et al., 2011; Toledo et al., 2012).

Enolase has also been suggested to be a major immunogenic protein with antigenic properties, and could therefore be a suitable candidate vaccine for pathogen infection (Zhang et al., 2015; Mahana et al., 2016; Membrebe et al., 2016). Recently, enolase has been demonstrated satisfactory protective properties against Plasmodium spp. challenge, suggesting that the protein could act as a viable vaccine for malaria (Pal-Bhowmick et al., 2007; Zhang et al., 2016). As both diseases are blood-borne apicomplexan parasites, Plasmodium spp. and Babesia spp. express a variety of similar functional proteins, such as rhoptry-associated protein 1, and apical membrane antigen 1 (Brown et al., 1996; Jakobsen et al., 1996; Xu et al., 2000; Tonkin et al., 2013), which are then targeted by the host to mount immune responses and protect against parasitic infection. One of the Babesia genus, *Babesia microti*, is the primary causative agent of human babesiosis in many regions worldwide. According to the published genomic sequences of *B. microti*, a single enolase gene located on chromosome III has been identified (Cornillot et al., 2012). However, the function of the expressed protein has not been confirmed, and its role during parasite infection processes is not yet clear.

In this study, we report the cloning, recombinant expression, biological characteristics, and potentially protective properties of *B. microti* enolase (BmEno). We demonstrate that the surface expressed BmEno catalyzes 2-PGA dehydration to PEP, and likewise also contributes to human plasminogen activation. Additionally, recombinant BmEno demonstrated a partial protective role against *B. microti* infection. However, further research is needed in order to evaluate BmEno’s potential as a candidate vaccine to control babesiosis infection.

## Materials and Methods

### Ethics Statements

This study was carried out in strict accordance with the recommendations of the guidelines for the Care and Use of Laboratory Animals of the Laboratory Animal Ethics Committee of Xuzhou Medical University. The protocol was approved by the Laboratory Animal Ethics Committee of Xuzhou Medical University (Permit Number: 201547).

### Parasites and Animals

The *B. microti* strain (ATCC^®^ PRA-99^TM^) was provided by the National Institute of Parasitic Diseases, Chinese Center for Disease Control and Prevention (Shanghai, China), and maintained in our laboratory. The NOD-SCID (non-obese diabetic/severe combined immunodeficiency) mice were used as reservoirs for preserving the parasites and the percentage of parasitized RBCs was determined via Giemsa-stained thin blood films. Female Balb/c mice (4–6 weeks old) were intraperitoneally inoculated with 100 μl infected blood, which was collected, heparinized, and diluted with sterile saline to achieve a 30% RBC parasitic infection rate. Parasites were isolated once 50–60% of RBCs were infected. All mice were purchased from NBRI (Nanjing Biomedical Research Institute of Nanjing University, Nanjing, China) and maintained at the Animal Center of Xuzhou Medical University.

### Reagents

All reagents were purchased from Sigma–Aldrich (St. Louis, MO, USA) unless otherwise specified.

### Bioinformatics Analysis of the BmEno Gene and Predicted Protein

*Babesia microti* enolase nucleotide sequence BLAST searches against published genomic sequences of *B. microti* were performed with the NCBI nucleotide database search program (Cornillot et al., 2012). The enolase signature sequence and other domains were defined using the ScanProsite online software^1^, and the MotifScan online software^2^. Multiple-sequence alignments were built with the ClustalX program^3^.

### BmEno Cloning, Recombinant Expression, and Purification

Total RNA was isolated from *B. microti*-infected RBCs with TRIzol reagent (Invitrogen, Carlsbad, CA, USA) according to the manufacturer’s instructions. The gene fragment encoding the entire BmEno coding sequence was obtained using RT-PCR amplified with a One Step PrimeScript RT-PCR Kit (Takara, Dalian, China), and the primers: BmEnoF (5′-CAGGGATCCATGACAAAGATAATTAGCGC-3′) and BmEnoR (5′-CCGCTCGAGTTACTTGTTAAAAACTTTGC-3′). Primers contained *Bam*HI and *Xho*I restriction sites (underlined) to assist subsequent cloning into the expression vector pGEX-4T-2 (TransGen Biotech, Beijing, China), containing a GST tag to facilitate purification. The resulting plasmid was transformed into *Escherichia coli* BL21 Star (DE3) competent cells.

For recombinant expression, *E. coli* cells were grown to 0.5 OD_600_ at 37°C, and then induced overnight with 0.4 mM isopropyl-β-D-thiogalactopyranoside (IPTG) at 25°C. Cell cultures were harvested by centrifugation and the resulting cell pellet was resuspended in sonication buffer (1 × PBS, 0.1% TritonX-100, 1 mg/mL lysosome, 5 mM DTT, 0.5 mM PMSF, pH 7.4) and lysed on ice with a sonicator. Debris was then pelleted by centrifugation, and the supernatant (containing recombinant BmEno, rBmEno) was purified by an affinity chromatography system on a GSTrap 4B column following the manufacturer’s instructions (GE Healthcare, Pittsburgh, PA, USA). Thrombin was used to cut the GST tag from the purified protein, which was then analyzed by sodium dodecyl sulfate–polyacrylamide gel electrophoresis (SDS–PAGE) followed by Coomassie brilliant blue staining. The purified protein was quantitated, divided into aliquots, and stored at -80°C until used.

### Western Blot Analysis

To determine whether anti-*B. microti* antibody recognizes rBmEno, Western blot was performed. In brief, 2 μg of purified rBmEno were separated by SDS–PAGE, and then transferred to activated polyvinylidene difluoride (PVDF) membrane. Membranes were blocked with Tris-buffered saline-Tween 20 (TBST; 20 mM Tris-base, 150 mM NaCl, 0.05% (v/v) Tween 20, pH 7.5) containing 5% (w/v) dry non-fat milk powder for 3 h at room temperature. The membrane was then either incubated with mouse anti-*B. microti* serum or normal mouse serum (1:500 dilution) overnight at 4°C. Following three washes in TBST, the membrane was incubated with enhanced chemiluminescence (ECL)-goat alkaline phosphatase (AP)-conjugated anti-mouse IgG (1:2000 dilution) for 2 h at room temperature. Following additional washings, the membrane was stained with ECL substrate solution and reactions were detected by exposure to X-ray film.

### Generation of Mouse Antisera against rBmEno

To generate polyclonal antibodies against rBmEno, five female Balb/c mice (4–6 weeks old) were immunized three times at two-weekly intervals by subcutaneous injection with purified rBmEno protein (50 μg/mouse), mixed with MONTANIDE^TM^ ISA 70 VG adjuvant (Seppic, Puteaux, France). One week after the last immunization, mouse blood samples were isolated to generate anti-serum by centrifuging. Antibody titer was measured using an indirect enzyme-linked immunosorbent assay (ELISA), where each well of a 96-well plate was coated with 2 μg rBmEno diluted in 100 μl coating buffer (1.5 g/L Na_2_CO_3_, 2.93 g/L NaHCO_3_, pH 9.6). Serum aliquots were then stored at -20°C until use.

### Immunoprecipitation

To determine whether anti-rBmEno antibody recognizes native BmEno, immunoprecipitation was performed. Briefly, 2 mg of *B. microti*-infected or normal mouse RBC lysates were mixed with 10 μl anti-rBmEno antibody overnight at 4°C. Then 30 μl of a 1:2 A/G-Sepharose protein slurry was added for 2 h at 4°C. After three washes with PBS, the precipitate was mixed with SDS–PAGE sample buffer and boiled. Western blot was performed as described above.

### Indirect Immunofluorescence Assay

*Babesia microti*-infected RBCs were smeared on coverslips, and fixed with 100% methanol for 30 min at -20°C. Samples were then permeabilized with PBS containing 0.1% (v/v) Triton X-100 for 15 min at room temperature. Following three washes with PBS, the samples were blocked in BSA-PBS containing 5% (w/v) BSA for 30 min at room temperature. Samples were then incubated overnight with polyclonal mouse anti-rBmEno serum (1:20 dilution) at room temperature. After three washes, samples were incubated with FITC-conjugated goat anti-mouse IgG (1:50 dilution) for 1 h at room temperature. After three washes, coverslips were briefly swirled in distilled water, mounted with 6-diamidino-2-phenylindole (DAPI), and then sealed with nail polish. Finally, images were acquired and analyzed using a ZEISS LSM 880 confocal microscope system (Zeiss, Munich, Germany).

### Characterization of rBmEno Enzymatic Activity

The enzymatic activity of purified rBmEno was determined by direct monitoring of absorbance enhancement at 240 nm and 30°C using a Synergy HT spectrophotometer (Bio-Tek Instruments, Winooski, VT, USA) as previously described (Figueiredo et al., 2015). Briefly, the assay was performed in a 30°C preheated reaction buffer (100 mM HEPES buffer, 7.7 mM KCl, 10 mM MgSO4, pH 7.0) with 1 mM 2-PGA and different concentrations of purified rBmEno (2.5, 5, 10, and 20 ng/μl). The total reaction volume was 200 μl. Continuous readings were performed every 3 min for a total of 66 min. BSA was used as a negative control, and reactions were performed in triplicate.

The Michaelis–Menten constant (*K*_m_) for the glycolysis substrate 2-PGA was determined from initial reaction measurements at concentrations ranging from 1 to 10 mM. Reactions were initiated by the addition of purified rBmEno at 1 μg/reaction. *K*_m_ values were calculated using computerized non-linear regression analysis of the data fitted to the Michaelis–Menten equation using Graphpad Prism 5.0 (GraphPad Software, Inc., USA).

To determine the effects of pH and temperature on rBmEno activation, rBmEno activity was determined using a reaction buffer system covering a pH range of 2.0–12.0, and a temperature range of 4–90°C. Reactions were initiated by the addition of rBmEno (1 μg/reaction) diluted in the corresponding buffer. Continuous readings were carried out every 3 min for 66 min, and reactions were performed in triplicate.

### Plasminogen-binding Assay

Recombinant BmEno binding to plasminogen (PLG) was assayed by ELISA as previously described (Nogueira et al., 2012). In brief, each well of a 96-well plate was coated overnight at 4°C with 4 μg/well human PLG diluted in 100 μl PBS. Non-specific binding sites were blocked with 2% BSA in PBS for 2 h at 37°C. After three washes with PBST containing 0.05% (V/V) Tween 20, wells were incubated with 4 μg/well rBmEno for 2 h at 37°C; to analyze the role of lysine residues in enolase and PLG binding activity, 4 μg/well of rBmEno were added in the presence or absence of 0–100 mM ε-aminocaproic acid (ε-ACA, lysine analog). After three washes, wells were incubated with polyclonal mouse anti-rBmEno serum (1:200 dilution) for 1 h at 37°C. After another three washes, all wells were then incubated with goat AP-conjugated anti-mouse IgG (1:10,000 dilution) for 1 h at 37°C. Finally, tetramethylbenzidine substrate (TMB) was added, and reactions were stopped by the addition of 100 μl/well of 2 N H_2_SO_4_. Absorbance was read at 450 nm using a Bio-Tek plate reader (Bio-Tek Instruments, Winooski, VT, USA). BSA was used as a negative control, and each assay was performed in triplicate.

### Plasminogen Activation Assay

The assay was performed in 96-well plates as previously described (Floden et al., 2011). Briefly, each well was coated overnight with 4 μg rBmEno in 100 μl PBS at 4°C. After being blocked and washed as above, 4 μg/well of human PLG was added and incubated for 2 h at 37°C. Following three washes, 4 ng/well of human urokinase plasminogen activator (uPA) was added, and then the plasmin-specific substrate D-valyl-leucyl-lysine-*p*-nitroanilide dihydrochloride was added at a final concentration of 0.3 mM in PBS. Plates were incubated overnight at 37°C, and absorbance was read at 405 nm using a Bio-Tek plate reader. BSA was used as a negative control, and the assay was performed in triplicate.

### Active Immunization and Infection Analysis

Female Balb/c mice (4–6 weeks old) were immunized with adjuvant containing purified rBmEno protein (20 μg/mouse) as described above, and control group mice were vaccinated with PBS in adjuvant. Two weeks after the third boost, mice were intraperitoneally inoculated with *B. microti* as described above. Blood smears were performed every 2 days to detect the percentage of infected RBCs by visual examination and counting using a microscope. Mice were sacrificed 8 days after infection. Blood samples were collected and genomic DNA was isolated with the QIAamp DNA Blood Mini Kit (Qiagen, Hilden, Germany). *B. microti* burden in mouse blood was evaluated via real time quantitative RT-PCR (qRT-PCR) with the isolated DNA.

### Real Time Quantitative PCR

qPCR was performed using the SYBR^®^ Premix *Ex* Taq Kit (Takara, Dalian, China) and run on the Roche LightCycler^®^ 480 System following manufacturer’s instructions. *B. microti* was quantified using primers targeting the small-subunit rRNA gene and qPCR results were analyzed followed as previously described (Bloch et al., 2013). qPCRs were performed in triplicate.

### Statistical Analysis

Statistical analysis of enzyme activity, plasminogen binding, and activation of rBmEno was performed using one-way analysis of variance (ANOVA) and Tukey’s multiple comparison tests; statistical analysis of qPCR was performed using two-tailed Student’s *t*-tests. The values were presented as mean ± standard deviation (SD) and considered significant at *p* < 0.05, data analysis was performed with Graphpad Prism 5.0.

## Results

### *Babesia microti* Enolase

**FIGURE 1:**
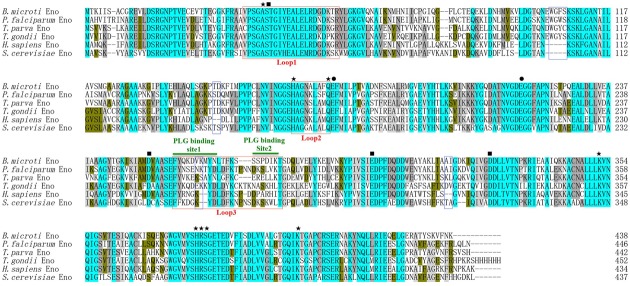
***Babesia microti* enolase (BmEno) bioinformatics analysis.** Comparison of enolase amino acid sequences from *Babesia microti* (XP_012649799), *Plasmodium falciparum* (AAA18634), *Theileria parva* (XP_764336), *Toxoplasma gondii* (3OTR_A), *Homo sapiens* (CAA36216), and *Streptococcus cerevisiae* (AOG56016) was performed with the ClustalX program. Residues interacting with 2-phospho-D- glycerate (2-PGA), residues involved in dehydration steps, and residues interacting with Mg^2+^ are marked by stars, circles, and rectangles, respectively. Catalytic loops and insertions are boxed. Plasminogen (PLG) binding sites are indicated with horizontal green lines.

An online search for *B. microti* genomic sequences identified one full-length cDNA (accession number: XM_012794345) comprising 1317 nucleotides encoding a 438 amino acid protein. This protein was predicted to be a soluble 47.5 kDa polypeptide with a pI of 5.98, displaying an enolase signature motif spanning residues 349-362 (L^349^-S^362^). With subsequent protein sequence analysis, the 2-PGA binding residues of BmEno, specifically Ala^40^, His^165^, Gln^173^, Lys^352^, His^380^, Arg^381^, Ser^382^, and Lys^403^ were conserved, and both Glu^174^ and Glu^217^ shared a proton during dehydration. In addition, Asp^252^, Glu^300^, and Asp^327^ were found to interact with an Mg^2+^ ion, and Ser^41^ bound the second Mg^2+^. Three catalytic loops of BmEno were predicted, loop1 (V^36^∼R^57^), loop2 (H^165^∼Q^173^), and loop3 (F^258^∼T^279^). BmEno includes pentapeptide (E^103^W^104^G^105^F^106^S^107^) and dipeptide insertions (V^263^K^264^), which are commonly known as plant-like insertions. Two predicted plasminogen-binding motifs were identified at F^258^∼N^267^ and S^273^∼K^278^, suggesting that BmEno could bind plasminogen (**Figure 1**). The protein sequence also carried phosphorylation and *N*-glycosylation sites, but no *trans*-membrane domains or signal peptides.

### rBmEno Expression, Purification, and Antigenic Characterization

**FIGURE 2:**
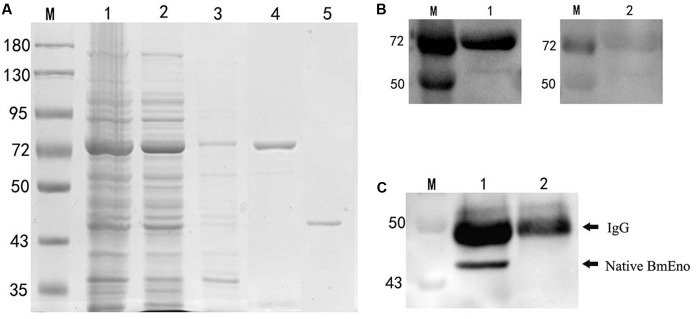
**Recombinant BmEno (rBmEno) expression, purification, and antigenic characterization. (A)** Sodium dodecyl sulfate–polyacrylamide gel electrophoresis (SDS–PAGE) analysis of rBmEno expression and purification followed by Coomassie blue staining. M: prestained protein ladder, Lane 1: total cellular proteins of pGEX-4T-2-BmEno BL21 (DE3) cells, Lane 2: supernatant of pGEX-4T-2-BmEno BL21 (DE3) cell lysate, Lane 3: sediment of pGEX-4T-2-BmEno BL21 (DE3) cell lysate, Lane 4: GST-tagged purified recombinant BmEno protein, Lane 5: purified recombinant BmEno protein. **(B)** Anti-*B. microti* serum recognized purified recombinant BmEno. M: prestained protein ladder, Lane 1: purified GST-tagged rBmEno proteins were immunoblotted with mouse polyclonal anti-*B. microti* serum, Lane 2: purified GST-tagged rBmEno proteins were immunoblotted with normal mouse serum. **(C)** Anti-rBmEno serum recognized native BmEno. M: prestained protein ladder, Lane 1: *B. microti*-infected red blood cell (RBC) lysates were immunoprecipitated with anti-rBmEno antibody, Lane 2: normal RBC lysates were unable to be immunoprecipitated with anti-rBmEno antibody.

Full-length enolase cDNA was amplified from the ATCC^®^ PRA-99^TM^ *B. microti* strain using specific primers, and then cloned in frame into the pGEX-4T-2 plasmid with a GST-tag domain as described in the “Materials and Methods.” rBmEno expression was induced in BL21 (DE3) *E. coli* with IPTG, and bacterial extracts were assessed by SDS–PAGE with Coomassie blue staining. Induced GST-tagged rBmEno protein migrated at approximately 73 kDa, with greater amounts observed in the supernatant than the sediment, whereas purified rBmEno produced a single 47.5 kDa band (**Figure 2A**).

To analyze whether anti-*B. microti* serum recognized purified recombinant BmEno, purified GST-tagged rBmEno was incubated with the mouse anti-*B. microti* serum. A strong band of approximately 73 kDa was observed in ECL-Western blot analysis (**Figure 2B**). Moreover, to determine whether anti-rBmEno antibody recognizes native BmEno, immunoprecipitation was performed, and native BmEno was immunoprecipitated with anti-rBmEno antibody (**Figure 2C**).

### Immunofluorescent Localization of BmEno in Intraerythrocytic *B. microti*

**FIGURE 3:**
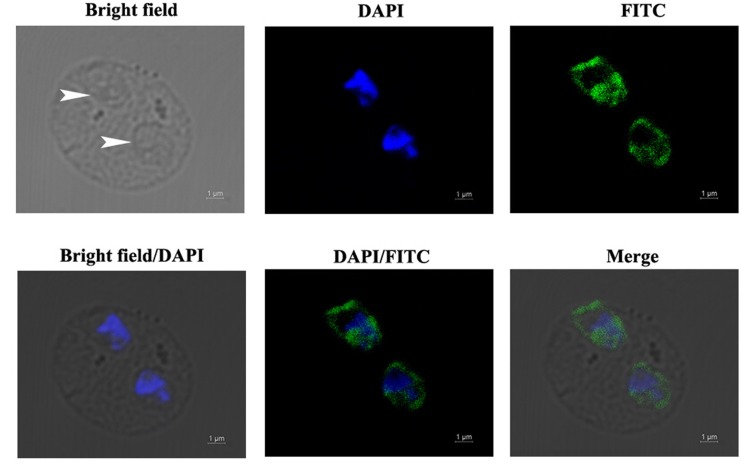
**Immunofluorescent localization of BmEno in intraerythrocytic *B. microti*.** Anti-rBmEno antibody reacted with native BmEno both in the cytoplasm and on the surface of *B. microti*. Bright field images show an RBC infected with two *B. microti* parasites (white arrows). Under fluorescent imaging, nuclei are stained blue with 6-diamidino-2-phenylindole (DAPI), and BmEno is stained green, indicating reaction with FITC-conjugated anti-mouse IgG anti-rBmEno antibody. Scale bar represents 1 μm.

The localization of BmEno in intraerythrocytic *B. microti* parasites was determined by immunofluorescence analysis using a mouse anti-rBmEno antibody. Following incubation with the anti-rBmEno antibody, intraerythrocytic parasites were counterstained with an FITC-conjugated anti-mouse IgG and nuclei were stained with DAPI. In **Figure 3**, parasitic nuclei stained blue with DAPI and specific green anti-rBmEno antibody reactivity was also observed. BmEno appeared to localize both in the cytoplasm and on the surface of *B. microti* (based on the observed parasite shapes).

### Characterization of rBmEno Enzymatic Activity

**FIGURE 4:**
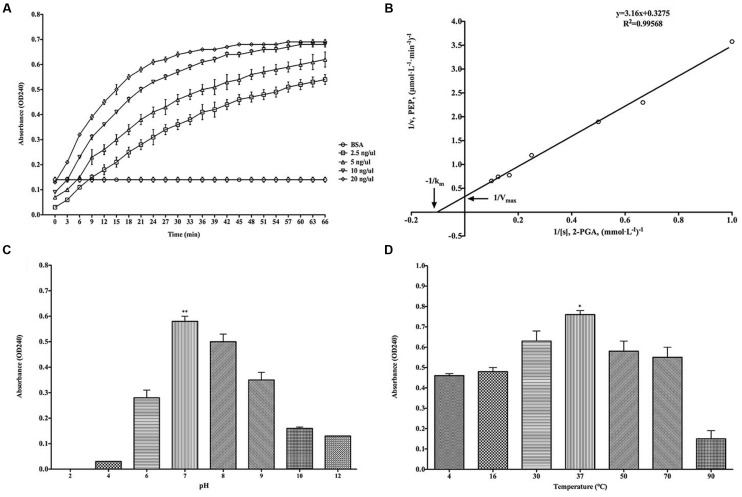
**Recombinant BmEno enzymatic activity and influencing factors. (A)** Effect of different rBmEno concentrations on enzymatic activity. **(B)** *K*_m_ and *V*_max_ for rBmEno were determined as 8.849 mM and 2.907 μmol/L/min, respectively, based on the Lineweaver–Burk plot (double-reciprocal plot). **(C)** rBmEno activity in a buffer system covering a 2–12 pH range, enzymatic activity was maximal at pH 7.0. **(D)** rBmEno activity in a buffer system covering the temperature range 4–90°C, enzymatic activity was maximal at 37°C. Relative activity of rBmEno was measured by OD240 absorbance. All data shown represent the mean ± SD from three independent experiments. Significant differences as compared to other conditions are denoted by ^∗^ for *p* < 0.05, and ^∗∗^ for *p* < 0.001.

The classical enzymatic activity of purified rBmEno was tested via its ability to convert 2-PGA to PEP. The catalytic activity of rBmEno increased with increasing concentrations from 2.5 to 20 ng/μl (**Figure 4A**). The kinetics of the conversion reaction revealed an 8.849 mM Michaelis constant (*K*_m_) and a maximum velocity (*V*_max_) of 2.907 μmol/L/min (**Figure 4B**). rBmEno enzyme activity was measured over a range of pH values, and the enzyme’s optimal pH was determined to fall within a neutral range, with maximal activity at pH 7.0 (**Figure 4C**). rBmEno enzyme activity was also assessed at varying temperatures and was found to have maximal activity at 37°C (**Figure 4D**).

### rBmEno Binds to Human Plasminogen and Enhances Its Activation

**FIGURE 5:**
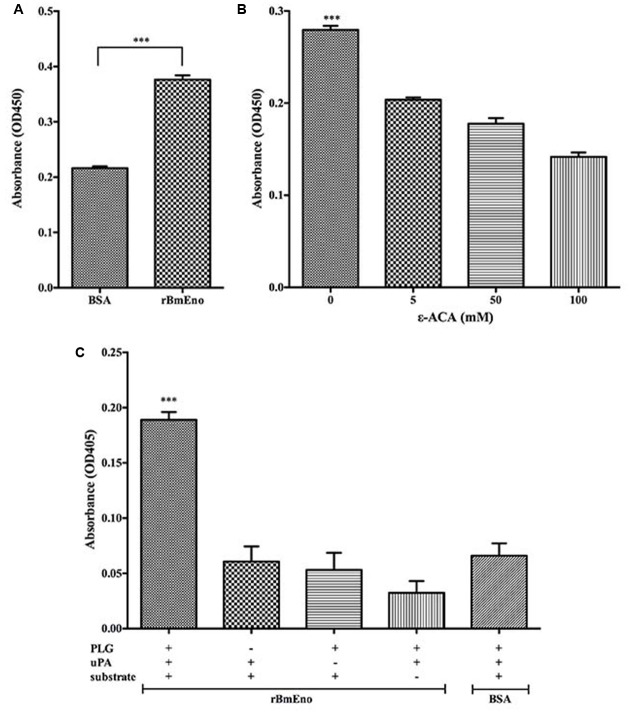
**Recombinant BmEno binds to human PLG and enhances its activation. (A)** rBmEno binding to immobilized human PLG protein was analyzed via enzyme-linked immunosorbent assay (ELISA), BSA was used as a negative control for non-specific binding. rBmEno binding activity was measured by absorbance at OD450. **(B)** rBmEno binding to immobilized PLG protein was analyzed by ELISA. rBmEno was added to PLG-coated wells in the presence or absence of 0–100 mM ε-aminocaproic acid (ε-ACA) rBmEno binding activity was measured by absorbance at OD450. **(C)** rBmEno-coated wells of microtiter plates were incubated with PLG, uPA, and/or a plasmin-specific chromogenic substrate, BSA was used as a negative control. Proteolytic activity was measured by absorbance at OD405. All data shown represent the mean ± SD from three independent experiments. Significant differences compared to control are denoted by ^∗∗∗^ for *p* < 0.0001.

To investigate rBmEno’s ability to bind human PLG, microtiter plates were coated with human PLG and BSA (negative control), and ELISA binding assays were performed. rBmEno was found to bind human PLG compared to BSA (*p* < 0.0001) (**Figure 5A**). To identify whether lysine residues play a role in enolase and PLG binding, the lysine analog ε-ACA was used as a binding competitor. ε-ACA significantly reduced the interaction between rBmEno and PLG (*p* < 0.0001) (**Figure 5B**). Enolase has been reported to enhance plasminogen to plasmin activation, thus to determine whether rBmEno also has this ability, microtiter plates were coated with rBmEno, blocked, incubated with PLG and uPA, and proteolytic activity measured using a plasmin-specific chromogenic substrate. rBmEno dramatically enhanced plasmin activation in the presence of uPA compared to the negative control BSA (*p* < 0.0001) (**Figure 5C**).

### Active rBmEno Immunization of Mice Evokes Partial Protective Immunity

**FIGURE 6:**
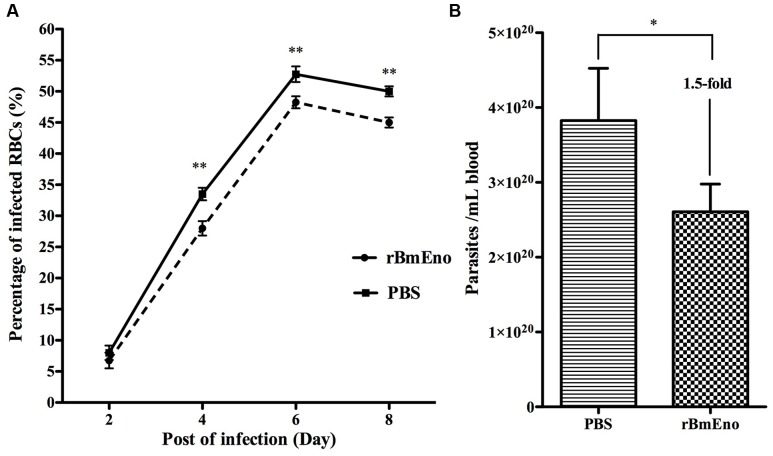
**Active immunization of mice with rBmEno evokes partial protective immunity. (A)** The percentage of infected RBCs was determined by counting the number of infected RBCs versus the total number of RBCs using blood smear with Giemsa staining, a minimum of 600 RBCs were counted and an RBC infected with multiple parasites was counted as a single infected cell. **(B)** The parasitic burden in mouse blood was evaluated with qPCR to detect the *B. microti* small-subunit rRNA gene. All data shown represent the mean ± SD from four independent mice. Significant differences compared to control are denoted by ^∗^ for *p* < 0.05, and ^∗∗^ for *p* < 0.001.

We have demonstrated that BmEno is expressed at the surface of *B. microti* parasites, thus we hypothesized that immunization of mice with rBmEno could elicit protective immunity and influence any subsequent *B. microti* infection. Therefore Balb/c mice (four animals/group) were immunized with purified rBmEno, while control mice were injected with PBS mixed with similar volume of adjuvant. Two weeks after the final immunization, mice were intraperitoneally inoculated with *B. microti*. Blood smears performed every 2 days after infection demonstrated that the percentage of *B. microti*-infected RBCs from protein-immunized mice was significantly lower than that from control mice after 4 days following parasite (*p* < 0.001) (**Figure 6A**). Additionally, qPCR showed that the parasitic blood burden was significantly decreased 1.5-fold in immunized mice after 8 days following parasite infection, than in controls (*p* < 0.05) (**Figure 6B**).

## Discussion

Enolase is an essential glycolytic enzyme in intracellular glucose metabolism, but is also referred to as a multifunctional protein, as it is also expressed on the pathogen surfaces (Ghosh and Jacobs-Lorena, 2011). In this study, we characterized a 47.5 kDa protein containing signature enolase motif residues, derived from the apicomplexan parasite *B. microti*. In order to evaluate the BmEno functional motif, bioinformatics sequence alignment analysis was performed with freely available sequence data. Both the 2-PGA binding residues and catalytic loops of BmEno were conserved when compared to *Streptococcus cerevisiae* yeast enolase and *Toxoplasma gondii* enolase (Zhang et al., 1997; Ruan et al., 2015) (**Figure 1**). The plasminogen-binding motif (FYDKERKVYD), first analyzed in *Streptococcus pneumoniae* surface enolase, was found to be required for proteolytic activity in the infected host (Bergmann et al., 2003). This motif is presented as FYQKDVKMYN in BmEno (**Figure 1**). The Plasmodium enolase contains an alternate plasminogen-binding motif (DKSLVK) (Ghosh et al., 2011), and the similar sequence SSPDIK can be identified in BmEno (**Figure 1**). BmEno also contains the plant-like pentapeptide (EWGFS) and dipeptide (VK) insertions (Dzierszinski et al., 2001). Deleting the EWGWS pentapeptide insertion from the *P. falciparum* enolase results in reduced *k*_cat_/*K*_m_ and dissociation into monomers (Vora et al., 2009). And finally, neither *trans*-membrane domains nor signal peptides were predicted in BmEno, thus confirming identification as an enolase-like structure (Pancholi, 2001; Diaz-Ramos et al., 2012). Taken together, BmEno contains catalytic residues, catalytic flexible loops, several insertions, and plasminogen-binding sites.

In order to biochemically characterize BmEno, we prepared recombinant BmEno protein in *E. coli* BL21 (DE3), and immunized Balb/c mice with purified rBmEno to generate anti-rBmEno serum. rBmEno was highly expressed as an IPTG-inducible soluble protein in BL21 (DE3), anti-rBmEno mouse serum recognized native BmEno, and mice were able to produce antibodies to BmEno during the natural course of infection (**Figure 2**). Taken together, these results indicate that enolase could be used as a potential diagnostic biomarker for babesiosis, as it has already been used for the diagnosis of numerous infectious diseases such as malaria or leishmaniasis (Sato et al., 2000; Duarte et al., 2015). However, its applicability in diagnostic tests should be fully investigated. Until recently, enolase was only recognized as an important cytosolic protein, however, new evidence confirms its presence on the surface of a variety of organisms (Pal-Bhowmick et al., 2007; Floden et al., 2011; Figueiredo et al., 2015). In the current study, our immunofluorescence results also confirmed that *B. microti* enolase is expressed in the cytosol and additionally on the organism surface (**Figure 3**).

As a cytoplasmic protein, enolase plays a pivotal role in glucose metabolism (Pancholi, 2001; Diaz-Ramos et al., 2012). Purified rBmEno was able to catalyze the conversion of 2-PGA to PEP in a reaction buffer with 10 mM Mg^2+^ at 30°C and pH 7.0. However, the enzymatic activity was influenced by both temperature and pH (**Figure 4**). Furthermore, rBmEno showed high *K*_m_ and *V*_max_ values compared to other pathogenic organisms during 2-PGA to PEP conversion (Bao et al., 2014; Figueiredo et al., 2015). This may be due to the fact that the parasite produces its energy mainly through anaerobic glycolysis during erythrocytic stages (Cornillot et al., 2012).

As a surface-exposed protein, the plasminogen receptor enolase can promote the conversion of the zymogen plasminogen to its active form, plasmin, in the presence of plasminogen activator (Floden et al., 2011; Ghosh et al., 2011; Nogueira et al., 2012; Toledo et al., 2012; Figueiredo et al., 2015). In other protozoan parasites such as *P. falciparum*, *Leishmania mexicana, and Trichomonas vaginalis*, enolase has been shown to bind to plasminogen (Vanegas et al., 2007; Mundodi et al., 2008; Bhowmick et al., 2009). Subsequently, our ELISA experiment to examine BmEno/plasminogen binding confirmed that rBmEno was also able to bind to human plasminogen (**Figure 5A**). It’s well known that lysine residues play an important role in the interaction of plasminogen receptors with their ligand (Ghosh and Jacobs-Lorena, 2011), and that competitive binding assays with lysine analogs such as ε-ACA have demonstrated that most bacterial and parasitic enolases do bind plasminogen through their lysine residues (Miles et al., 2005; Figuera et al., 2013; Miles and Parmer, 2013). Furthermore, in *S. pneumoniae* enolase, lysine residues have been identified in the “FYDKERKVYD” motif (Bergmann et al., 2003), similar to the “FYQKDVKMYN” BmEno plasminogen-binding domain predicted by bioinformatics analysis. Our competitive binding experiments with ε-ACA significantly inhibited rBmEno plasminogen binding (**Figure 5B**), thereby confirming that lysine residues play an essential role in BmEno/plasminogen binding.

The active form of the mammalian liver enzyme plasminogen is plasmin, a serine protease that plays a key role in degrading the target host cell extracellular matrix during infection with pathogenic organisms (Lahteenmaki et al., 2005; Gonzalez-Miguel et al., 2016). Plasminogen activation also requires tissue/uPA, and it has also been reported that surface expressed enolase can enhance the activation process (Floden et al., 2011; Ghosh et al., 2011; Nogueira et al., 2012; Toledo et al., 2012; Figueiredo et al., 2015). We showed that rBmEno dramatically enhanced plasmin activation in the presence of urokinase plasminogen activator (**Figure 5C**). Interestingly, *in vitro* studies have shown that *P. falciparum* enolase antibody was able to block merozoite invasion of RBCs (Pal-Bhowmick et al., 2007) suggesting that enolase plays an unknown role in the RBC invasion process. Here, we hypothesized that apicomplexan parasites utilize surface-expressed enolase to promote plasmin generation, which then degrades RBC membrane sections, enabling rapid RBC invasion. Moreover, apicomplexan parasites could also utilize the ability of surface-exposed enolase to bind plasminogen to invade vector gut epithelium (Ghosh et al., 2011).

Moreover, enolase may also be an important candidate antigen for vaccines against pathogen infection (Pal-Bhowmick et al., 2007; Zhang et al., 2015, 2016; Mahana et al., 2016; Membrebe et al., 2016). It has been reported that mice immunized with r-Pfen antigen were strongly protected against infection when challenged with mouse malarial parasite *Plasmodium yoelii* (Pal-Bhowmick et al., 2007), therefore we also tested the protective properties of *B. microti* enolase. Our immunization experiments demonstrated that enolase antibodies slightly but significantly reduced both parasitemia and parasite levels in murine blood (**Figure 6**). Such variable protective properties may be due to varying quality of immunized protein. Furthermore, each mouse was only immunized with 20 μg rBmEno whereas 100 μg protein was used to immunize each mouse in the r-Pfen protection test. Further research is now required in order to evaluate BmEno’s potential as a candidate vaccine to control babesiosis infection.

## Conclusion

Our results demonstrate that the BmEno gene encodes a classical 47.5 kDa enolase enzyme, which possesses a highly conserved active site and plasminogen-binding domains. BmEno is internally expressed and likely plays a role in glycolysis, however, it also catalyzes the reversible dehydration of 2-PGA to yield PEP. Furthermore, BmEno is also expressed at the external surface parasite surface, where it can bind to and enhance the activation of plasminogen. We have also confirmed that lysine residues play an important role in BmEno and plasminogen interaction. Moreover, BmEno could evoke a partial protective response against *B. microti* infection, thus BmEno’s potential as a candidate vaccine to control babesiosis infection should be evaluated in the future. In summary, this detailed first characterization of Babesia enolase improves our understanding of host cell invasion mechanisms by this intraerythrocytic pathogenic parasite.

## Author Contributions

Conceived and designed the experiments: XL, XG, and KZ. Performed the experiments: XL, CZ, and XG. Analyzed the data: XL, CZ, and XG. Contributed reagents/materials/analysis tools: XL, CZ, and JC. Wrote the paper: XL and KZ. All authors read and approved the final version of the manuscript.

## Conflict of Interest Statement

The authors declare that the research was conducted in the absence of any commercial or financial relationships that could be construed as a potential conflict of interest.

## Footnotes

1 http://prosite.expasy.org/scanprosite/

2 http://myhits.isb-sib.ch/cgi-bin/motif_scan

3 http://www.ebi.ac.uk/Tools/msa/clustalo/
